# Supplementary material for: cGAS and STING in Host Myeloid Cells Are Essential for Effective Cyclophosphamide Treatment of Advanced Breast Cancer
Source: Cancers (Basel). 2025 Mar 28;17(7):1130. doi: 10.3390/cancers17071130 (PMC11987962; doi:10.3390/cancers17071130)
Supplement: Supplementary file 1 [file cancers-17-01130-s001.zip › cancers-3502175-supplementary.pdf]

Supplementary Materials: cGAS and STING in Host Myeloid Cells Are Essential to Effective Cyclophosphamide Treatment of Advanced Breast Cancer

Yein-Gei Lai, Hao-Ting Liao, Yung-Hsiang Chen, Shih-Wen Huang, Yae-Huei Liou, Zhen-Qi Wu and Nan-Shih Liao

Table S1. Antibodies used for flow cytometry analysis of cells dissociated from tumor tissue.

Protein	Vendor	Catalog Number/PRID	Titer
Anti-mouse CD45.2 BUV395 (mouse monoclonal)	BD Biosciences	Cat#564616, RRID:AB_2738867	(1:100)
Anti-mouse CD19 Brilliant Ultra Violet™ 615 (Rat monoclonal)	eBioscience	Cat#366-0193-82, RRID:AB_2925404	(1:100)
Anti-mouse TCRb PE/Dazzle™ 594 (Armenian Hamster monoclonal)	BioLegend	Cat#109240, RRID: AB_2565654	(1:100)
Anti-mouse CD4 APC/Fire™ 810 (Rat monoclonal)	BioLegend	Cat#100480, RRID: AB_2860583	(1:400)
Anti-mouse CD8a Super Bright™ 645 (Rat monoclonal)	eBioscience	Cat#64-0081-82, RRID:AB_2662353	(1:100)
Anti-mouse CD11b BUV805 (Rat monoclonal)	BD Biosciences	Cat#741934, RRID: AB_2871303	(1:400)
Anti-mouse CCR7 Alexa Fluor® 647 (Rat monoclonal)	BioLegend	Cat#120109, RRID:AB_389235	(1:50)
Anti-mouse NK1.1 PerCP/Cyanine5.5 (Mouse monoclonal)	BioLegend	Cat#108728, RRID: AB_2132705	(1:50)
Anti-mouse PD-1 eFluor™ 450 (Rat monoclonal)	eBioscience	Cat#48-9981-82, RRID:AB_11150068	(1:50)
Anti-mouse/human CD44 Brilliant Violet 510 (Rat monoclonal)	BioLegend	Cat#103044, RRID: AB_2561391	(1:80)
Anti-mouse CD62L BV711 (Rat monoclonal)	BD Biosciences	Cat#:740660	(1:800)
Anti-mouseTim-3 PE/Cyanine7 (Rat monoclonal)	BioLegend	Cat#119716, RRID: AB_2571932	(1:200)
Anti-mouse Ly108 BUV661 (Mouse monoclonal)	BD Biosciences	Cat#741679, RRID:AB_2871064	(1:200)
Anti-mouse Lag-3 Brilliant Violet 785 (Rat monoclonal)	BioLegend	Cat#:]125219, RRID: AB_2566571	(1:25)
Anti-mouse Foxp3 PE-Cyanine5 (Rat monoclonal)	eBioscience	Cat#15-5773-82, RRID: AB_468806	(1:100)
Anti-human/mouse Granzyme B PE (Mouse monoclonal)	BioLegend	Cat#372208, RRID: AB_2687031	(1:50)

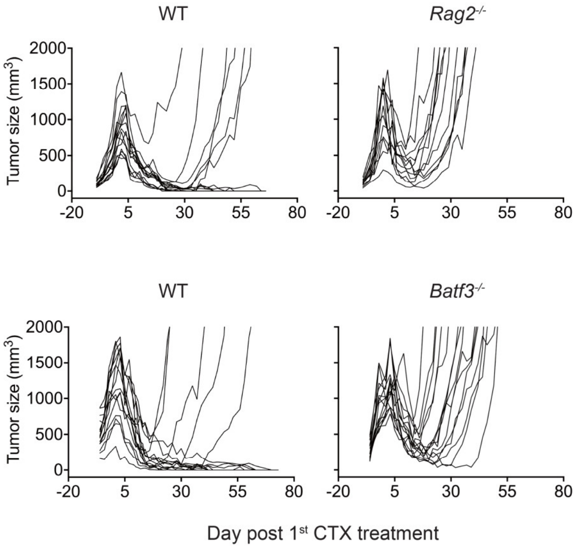

Figure S1. Tumor size curves of individual mouse in Figures 1B and 1D. Upper panels depict *Rag2*$^{-/-}$ (n = 12) and WT (n = 14) mice in Figure 1B. Lower panels depict *Batf3*$^{-/-}$ (n = 15) and WT (n = 15) mice in Figure 1D.

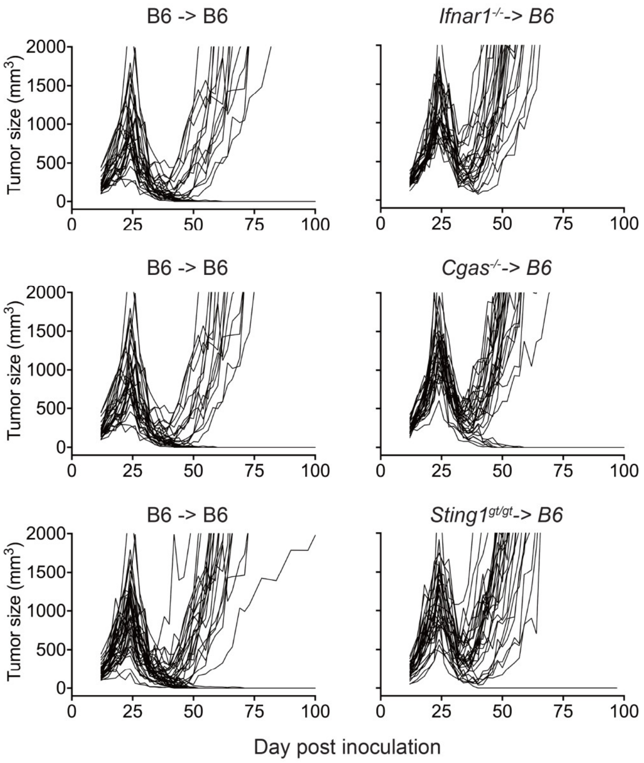

Figure S2. Tumor size curves of individual BM chimeric mouse in Figure 2.

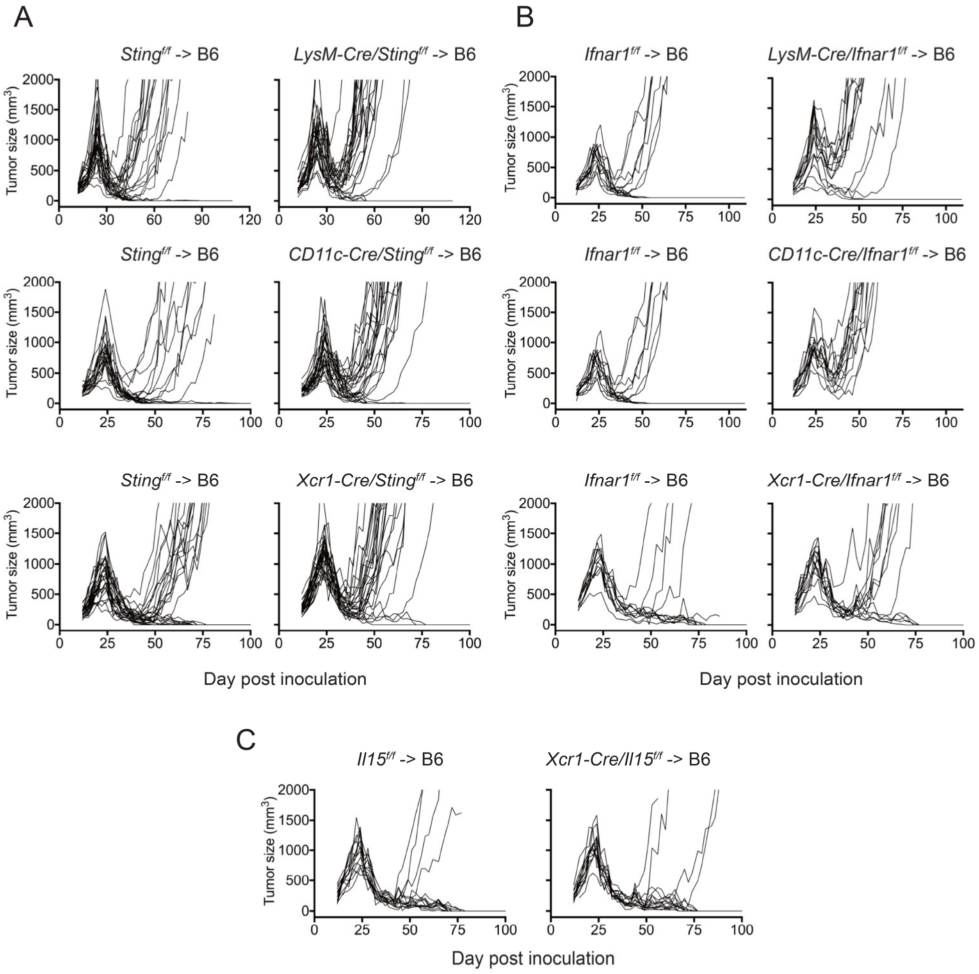

Figure S3. Tumor size curves of individual BM chimeric mouse in Figure 3. A. *LysM*$^{cre/+}$*Sting1*$^{f/f}$ → B6, *CD11c*$^{cre/+}$*Sting1*$^{f/f}$ → B6, and *XCR1*$^{cre/+}$*Sting1*$^{f/f}$ → B6 BM chimeric mice. B. *LysM*$^{cre/+}$*IFNar1*$^{f/f}$ → B6, *CD11c*$^{cre/+}$*IFNar1*$^{f/f}$ → B6, and *XCR1*$^{cre/+}$*IFNar1*$^{f/f}$ → B6 BM chimeric mice. C. *Xcr1*$^{cre/+}$*Il15*$^{f/f}$ → B6 and *Il15*$^{f/f}$ → B6 BM chimeric mice.

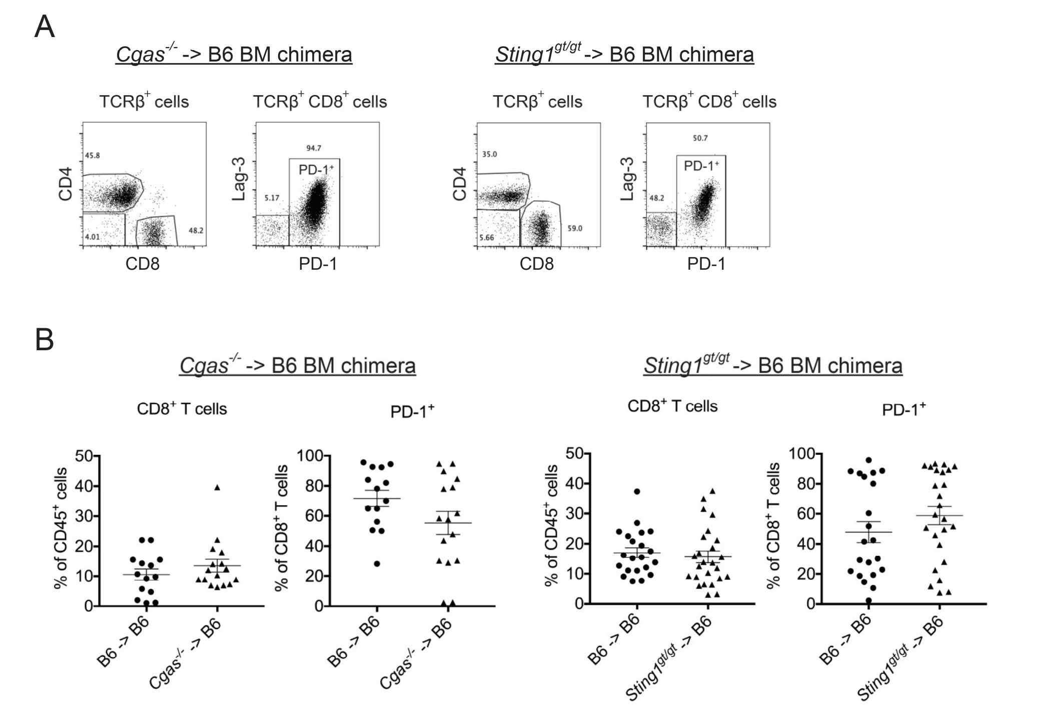

Figure S4. Analysis of intratumoral CD8$^+$ T cells in BM chimera in Figure 4A. A. Strategy to gate PD-1$^+$CD8$^+$ cells from TCRβ$^+$ tumor-infiltrated cells. The plots are from representative *Cgas*$^{-/-}$ → B6 and *Sting1*$^{gt/gt}$ → B6 chimeras. B. The proportion of CD8$^+$ T cells among CD45$^+$ cells and of PD-1$^+$ cells among CD8$^+$ cells from tumors of indicated BM chimeras. Data have been compiled from two independent experiments and the mean ± SEM of all samples in each group is presented. Each symbol in the graph represents 1-4 mice for B6 → B6 chimeras or one mouse for the indicated KO → B6 chimeric group.

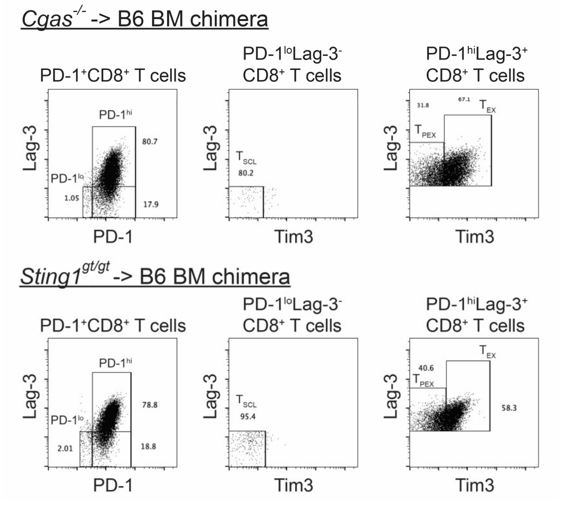

Figure S5. Gating strategy for intratumoral CD8$^+$ T$_{SCL}$, T$_{PEX}$ and T$_{EX}$ cells in Figure 4D. Strategy to gate T$_{SCL}$ cells from PD1^{lo}Lag-3$^-$ CD8$^+$ T cells, and to gate T$_{PEX}$ and T$_{EX}$ cells from PD1^{hi}Lag-3$^+$ CD8$^+$ T cells. Data are from representative *Cgas*$^{-/-}$ → B6 and *Sting1*$^{gt/gt}$ → B6 BM chimeras.
